# Intracytoplasmic sperm injection for non-male factor infertility does not improve cumulative live birth rate: a Canadian assisted reproductive technologies registry (CARTR Plus) descriptive study

**DOI:** 10.3389/frph.2026.1822305

**Published:** 2026-04-29

**Authors:** Z. M. Ferraro, L. Meng, K. E. Liu, E. M. Greenblatt, M. A. Russo

**Affiliations:** 1 Department of Obstetrics and Gynaecology, University of Toronto, Toronto, Ontario, Canada; 2 Mount Sinai Fertility, Mount Sinai Hospital, Toronto, Ontario, Canada; 3 Better Outcomes Registry & Network (BORN), Centre for Practice-Changing Research Building, Ottawa, Ontario, Canada

**Keywords:** non-male factor infertility, ICSI, cumulative live birth rate, fertilization rate, IVF

## Abstract

**Purpose:**

To determine whether intracytoplasmic sperm injection (ICSI) improves cumulative live birth rates (cLBR) compared with conventional IVF among patients with non–male factor infertility.

**Methods:**

This descriptive retrospective cohort study used linked Canadian Assisted Reproductive Technologies Registry (CARTR Plus) and Better Outcomes Registry & Network (BORN) data from 2013 to 2022. Outcomes were compared by insemination technique (IVF vs. ICSI). The primary outcome was cLBR per retrieval; secondary outcomes included normal fertilization (2PN), fertilization rate, utilizable embryos per retrieval, and cycles without live birth.

**Results:**

Among 140,252 retrieval cycles (35,251 IVF; 105,001 ICSI), cLBR was higher with conventional IVF (38.5% vs. 36.3%, *p* < 0.001). Oocyte yield was similar, although more oocytes were inseminated with IVF. Fertilization rates were slightly higher with ICSI, but normal fertilization and utilizable embryos per retrieval were comparable.

**Conclusions:**

In non–male factor infertility, ICSI does not improve cumulative live birth outcomes compared with conventional IVF.

## Introduction

To achieve pregnancy, many couples turn to assisted reproductive technologies (ARTs) including *in vitro* fertilization (IVF) with or without intracytoplasmic sperm injection (ICSI). Historically, ICSI was introduced to improve fertilization in couples with male factor infertility and for those with prior fertilization failure during an IVF cycle with normal semen parameters (1, 2). Since its advent, the use of ICSI has increased disproportionately relative to the number of couples with a male-factor infertility diagnosis, which has remained stable (3). The use of ICSI in couples with normal semen parameters suggests that this method of insemination is being used despite a lack of evidence of a beneficial effect over conventional IVF (cIVF) in this population (4, 5). The American Society for Reproductive Medicine (ASRM) guideline also described the ongoing lack of data to support the routine use of ICSI in non-male factor infertility (ASRM 2020) despite it being the most common method of fertilization (6).

A recent Cochrane review that included data from three randomized controlled trials (RCTs) with a total of 1539 couples compared insemination techniques in males presenting with normal total sperm count and motility, concluded that neither method was superior with respect to achieving a live birth or in the incidence of adverse events (e.g., multiple pregnancy, ectopic pregnancy, pre-eclampsia and/or prematurity). Secondary outcomes including clinical pregnancy, viable intrauterine pregnancy or miscarriage did not differ by insemination technique either (5).

Cumulative live birth rate (cLBR) is considered by many the gold standard when measuring treatment success as it allows for inclusion of fresh and frozen embryo transfers and resulting live births from a single stimulation cycle. Moreover, it has been argued that cLBR may be less in ICSI cycles, as only mature oocytes are inseminated, whereas in cIVF oocytes across the maturation spectrum can develop into embryos, thereby increasing the pool of potential embryos in development (7, 8). The use of cLBR can also serve as a more valuable metric for patient counseling, particularly when it comes to family planning. Although several retrospective studies and RCTs have evaluated the impact of ICSI in non-male factor infertility on live birth rate, only a few have reported on cLBR in a non-male factor infertility population (8–11).

This is the largest Canadian study that sought to evaluate the impact of insemination technique on cLBR in patients with non-male factor infertility.

## Methods

Since 2013, the Canadian Assisted Reproductive Technologies Registry (CARTR) Plus database has been used to monitor trends, performance, and outcomes of IVF treatment in Canada. In Ontario, in addition to IVF treatment cycles details, pregnancies from ART cycles are directly linked within the Better Outcomes Registry & Network (BORN) Information System, which contains information on birth outcomes (12). In the rest of Canada, individual clinics enter birth outcome data once available from patients or provincial birth records. All participating clinics anonymously submit data for all patients undergoing ART including cycle details and outcomes. The CARTR Plus database has been previously validated (13) and the data we present within is congruent with the CARTR Plus annual report (14).

**Figure 1:**
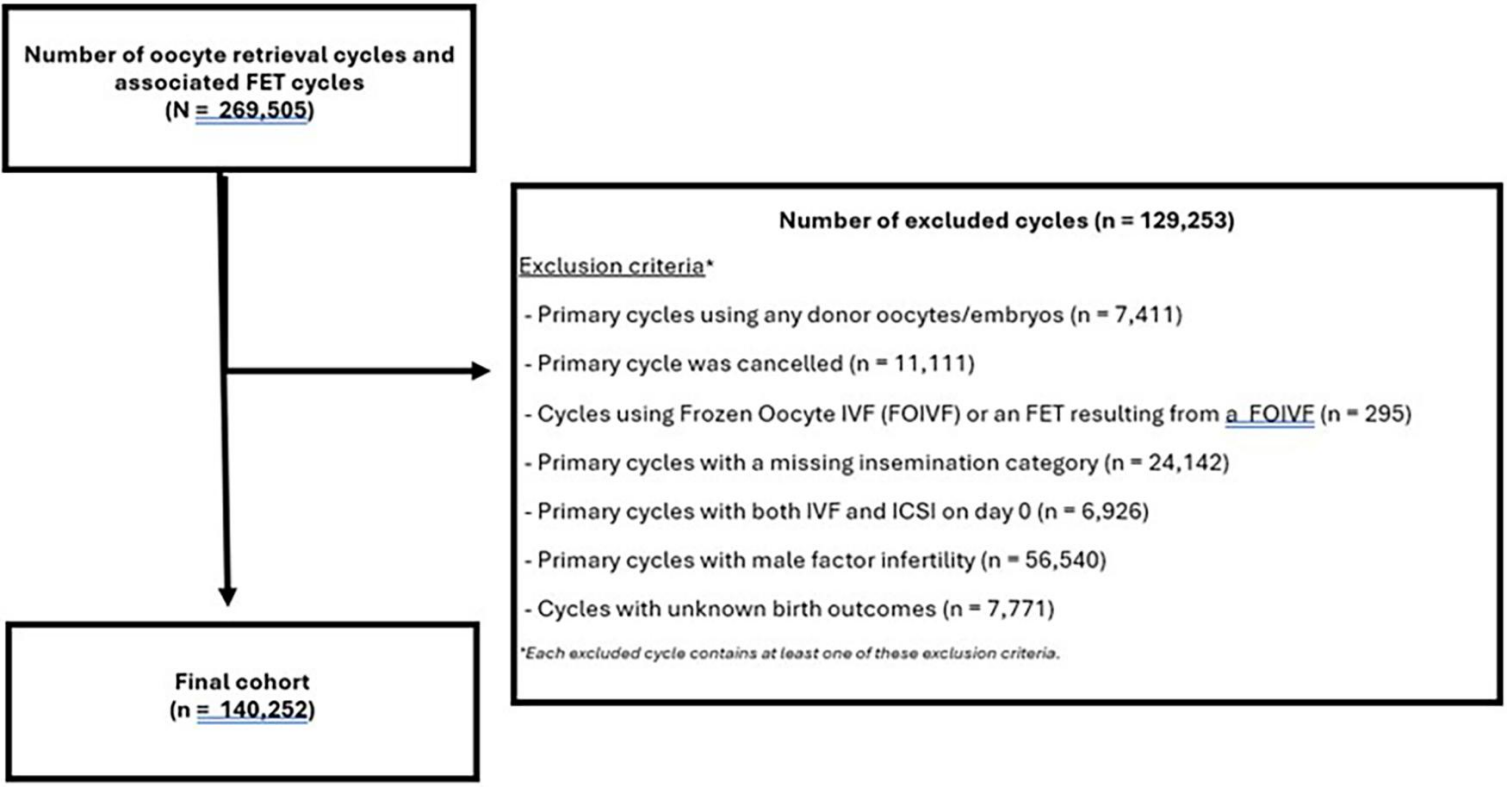
Study flow diagram. January 1, 2013-December 31, 2022.

Using the CARTR Plus database, we conducted a descriptive retrospective cohort study of all Canadian IVF fresh and frozen-thaw embryo transfer (ET) cycles with non-male factor infertility from January 1, 2013 to December 31, 2022 based on retrieval cycles. During this period, data was available for 35 Canadian ART clinics which includes all but two clinics in Canada (i.e., 95% of clinics represented). We excluded primary cycles using donor oocytes/embryos; cancelled cycles that did not have ovum pick up; cycles using previously frozen oocytes; primary cycles with missing insemination technique; primary cycles using a split of both IVF and ICSI; primary cycles with male factor infertility (based on abnormal semen parameters); cycles with missing birth outcomes and cycles with no associated fresh or FET cycles within a year of the retrieval date (Figure 1). Of note, it is the responsibility of individual clinics reporting to CARTR to correctly diagnose male factor as a subcategory of infertility, which is commonly done using the WHO criteria (15). Any abnormality in at least one of sperm concentration, motility, or morphology defines male factor and were thus excluded. Of note, isolated teratospermia is not an indication for ICSI and does not impact fertilization or live birth rates (2). Figure 1 highlights the 129 253 excluded cycles, and each cycle contains at least one of these exclusion criteria.

The primary outcome was cumulative live birth rate (cLBR), defined as the number of retrieval cycles resulting in at least one live birth within one year of the transfer, expressed as a percentage of all retrievals that had at least one autologous fresh or frozen embryo transfer within one year of the associated retrieval. Secondary outcomes included normal fertilization (2PN), fertilization rate (2PN/oocytes inseminated), the average number of utilizable embryos per retrieval (i.e., embryos transferred fresh and those frozen), and the number of individual cycles reporting no live birth (includes cycles that did not have an embryo transfer, negative pregnancy tests, chemical pregnancies, miscarriages). Characteristics of the study population are presented for total fresh cycles to avoid duplication of patients who went on to have additional FET cycles and cLBR treatment outcomes represent 35 of 37 clinics in Canada that submitted information to CARTR-BORN.

For both IVF and ICSI cycles, we obtained the total number of retrieval cycles resulting in at least one live birth within one year of the associated transfer. A live birth was defined as a baby born after 20 weeks gestation. Next, using a standardized approach (16), we obtained the total number of retrievals that had at least one ET within one year of the retrieval. Cumulative live birth rates are expressed as a percentage for retrieval cycles started from 2013 to 2022 and any associated fresh or frozen embryo transfer within 1 year of oocyte retrieval.

Our sample size calculation was based on previous data published in a non-male factor population (Boulet, 20215), which found a LBR of 36.5% with ICSI compared to 39.2% with IVF. If we assume an alpha of 0.05 and Power of 0.8, we need 5064 retrievals in each group, for a total of 10,128 retrieval cycles. Our sample size of 140 252 cycles, therefore, has greater power to detect potential differences between insemination techniques. Descriptive comparisons between conventional IVF and ICSI groups were assessed. Chi-square tests were used for categorical variables and Wilcoxon Two-sample tests were used for continuous variables to calculate *p*-values between the two groups. Data analysis was performed using SAS 9.4 software (SAS Institute, North Carolina, USA). Ethics approval was obtained from the Mount Sinai Hospital Research Ethics Board (MSH REB 21-0011-C).

## Results

The present study includes a total of 140,252 retrievals in patient(s) with non-male factor infertility, of which 35,251 (25.1%) utilized cIVF and 105,001 (74.9%) used ICSI as their insemination technique. Our primary outcome, cLBR, was greater in the IVF group (IVF 38.5% vs. ICSI 36.3%; *p* < 0.001) and based on all fresh and frozen embryo transfers that occurred within 1 year of the retrieval (Table 1).

**Table 1:** Cumulative live birth rates between IVF and ICSI.

		IVF			ICSI		
Year	Number of cycles resulting in at least one live birth	Number of cycles that had at least one ET within one year of the retrieval	Cumulative live birth rate	Number of cycles resulting in at least one live birth	Number of cycles that had at least one ET within one year of the retrieval	Cumulative live birth rate	*P* value
2013-2022	n	N	%	n	N	%	
Canada	7597	19727	38.5	17642	48565	36.3	<0.00001

Characteristics of the study population are presented for total fresh cycles to avoid duplication of patients who went on to have additional FET cycles (Table 2). Mean age of the sperm provider [IVF: 37.31 years (+/- 5.53) vs. ICSI: 38.28 years (+/- 5.94)] and of the oocyte provider [IVF: 35.77 years (+/-4.39) vs. ICSI: 36.41years (+/−4.37) was higher in the ICSI group. Most cycles (65.5%) were in women 35 years old and older. Of the 129,253 cycles excluded (Figure 1), there were 56,540 (∼21%) primary cycles with a male factor diagnosis. With respect to parity, most patients did not have a prior live birth [IVF: 82.75% vs. ICSI: 87.52%]. The average number of IVF cycles per patient were also similar [IVF: 1.28 (0.69) vs. ICSI: 1.40 (0.85)] (Table 2).

**Table 2:** Characteristics of study population. January 1, 2013-December 31, 2022.

Patient and IVF Cycle Characteristics	IVF		ICSI		*p*-Value
	(*n* = 24,413)		(*n* = 70,528)		
	*n*	(%)	*n*	(%)	
Age of sperm provider (years)					
Missing	4,332	17.74	19,592	27.78	
Mean (SD)	37.31 (5.53)		38.28 (5.94)		<.0001
Age of oocyte provider (years)					<.0001
0–34	9,368	38.37	23,332	33.08	
35–37	5,846	23.95	16,426	23.29	
38–40	5,348	21.91	16,983	24.08	
41–42	2,794	11.44	9,569	13.57	
43+	1,057	4.33	4,218	5.98	
Mean (SD)	35.77 4.39)		36.41 (4.37)		<.0001
BMI of embryo recipient					<.0001
<18.5	222	0.91	324	0.46	
18.5–25	4,652	19.06	5,749	8.15	
25–30	2,276	9.32	2,677	3.80	
30+	1,665	6.82	1,923	2.73	
Missing	15,598	63.89	59,855	84.87	
Mean (SD)	25.63 5.61)		25.41 (5.61)		0.0013
Prior births (parity)					<.0001
0	20,201	82.75	61,726	87.52	
1	3,471	14.22	7,324	10.38	
2+	741	3.04	1,477	2.09	
Missing Data	0	0	<6	<6	
Reason for Treatment					
Endometriosis	2,582	10.58	5,801	8.23	<.0001
Tubal factor	5,093	20.86	6,425	9.11	<.0001
Diminished ovarian reserve	4,864	19.92	15,649	22.19	<.0001
Unexplained Infertility	7,241	29.66	23,662	33.55	<.0001
Intent to perform PGT-A	2,202	9.02	18,034	25.57	<.0001
Poly Cystic Ovarian Syndrome + Other ovulatory disorders	124	0.51	182	0.26	<.0001
Other	3,718	15.23	13,720	19.45	<.0001
Missing Data	0	0	<6	<6	
Type of Stimulation Protocol					<.0001
Antagonist	16,557	67.82	55,293	78.40	
Flare agonist	845	3.46	3,293	4.67	
IVM protocol	< 6	S	164	0.23	
Long agonist	3,100	12.70	5,740	8.14	
Microdose flare agonist	1,475	6.04	2,963	4.20	
Mild stimulation	270	1.11	1,017	1.44	
Modified natural cycle	235	0.96	367	0.52	
Natural cycle	704	2.88	973	1.38	
Other	1,225	5.02	715	1.01	
Missing Data	0	0	<6	<6	
Trigger Medication					<.0001
GnRH agonist	2,164	8.86	6,847	9.71	
hCG	1,279	5.24	4,814	6.83	
hCG + GnRH agonist	566	2.32	4,314	6.12	
rec-hCG	7,275	29.80	23,928	33.93	
rec-hCG + GnRH agonist	518	2.12	2,365	3.35	
u-hCG	10,686	43.77	18,016	25.54	
u-hCG + GnRH agonist	486	1.99	2,210	3.13	
Other	765	3.13	2,406	3.41	
None	420	1.72	4,751	6.74	
Missing Data	254	1.04	877	1.24	
Prior fresh cycles for that patient					<.0001
0	16,354	66.99	42,556	60.34	
1	5,025	20.58	16,730	23.72	
2+	3,034	12.43	11,241	15.94	
Missing Data	0	0	< 6	< 6	
Average maximum number of IVF cycles per patient					<.0001
Mean (SD)	1.28 (0.69)		1.40 (0.85)		
AMH of oocyte provider (ng/mL)					<.0001
Mean (SD)	4.46 (7.06)		5.31 (8.26)		
Total dose of FSH					<.0001
Mean (SD)	3,159.88 (1,749.04)		3,287.00 (1,711.11)		
Estradiol level at trigger					<.0001
Mean (SD)	7,959.35 (6,253.22)		8,339.51 (6,804.68)		
Progesterone level at trigger					<.0001
Mean (SD)	4.51 (8.47)		6.45 (11.81)		
Number of Days of Gonadotropins					<.0001
Mean (SD)	10.86 (2.94)		11.17 (3.50)		
Number of small, growing follicles (<15 mm) at trigger					<.0001
Mean (SD)	5.11 (6.45)		4.97 (6.91)		
Number of large, growing follicles (>15 mm) at trigger					<.0001
Mean (SD)	6.81 (5.85)		6.35 (6.00)		
Number of oocytes retrieved					0.0101
Mean (SD)	10.95 (7.62)		10.93 (7.84)		
Number of metaphase II oocytes*					<.0001
Mean (SD)	7.45 (6.78)		7.88 (6.29)		
Semen Volume (mL)					<.0001
Mean (SD)	2.92 (1.43)		2.79 (1.48)		
Sperm Count (million/mL)					
Mean (SD)	78.54 (60.58)		65.84 (61.45)		<.0001
Sperm Motility (%)					
Mean (SD)	58.85 (16.07)		51.46 (18.64)		<.0001

The IVF group had a greater proportion of patient(s) with a diagnosis of endometriosis, tubal factor, and PCOS, whereas the ICSI group had a greater proportion of patient(s) diagnosed with diminished ovarian reserve, unexplained infertility, and/or intent to perform PGTA (Table 2). The type of stimulation protocol used was statistically different between groups, with the antagonist protocol accounting for 75.68% of total fresh IVF/ICSI cycles [IVF 67.8% vs. ICSI 78.4% (Table 2)].

Anti-mullerian hormone (AMH) levels were lower in the IVF group (mean ± SD) 4.46 ng/mL ±7.06 compared to ICSI 5.31 ng/mL ± 8.26. Total dose of FSH, estradiol and progesterone levels on trigger day, number of days of gonadotropins, number of small (<15 mm) and large (>15 mm) growing follicles at trigger day were also similar between IVF and ICSI groups (Table 2).

Number of oocytes retrieved (mean ± SD) was similar between IVF (10.95 ± 7.62) and ICSI 10.93 ± 7.84. The number of metaphase II oocytes injected via ICSI and the number of MII oocytes on day 1 after conventional IVF were also similar between groups at ∼7 (Table 2). Semen parameters were normal in both groups (Table 2). The average (SD) number of oocytes inseminated/injected was greater in the IVF group [10.67(7.49) vs. ICSI 8.40(6.16), *p* < 0.001]. Normal fertilization (2PN) was similar between groups when stratified by both age of the oocyte and sperm provider. However, as expected due to fewer oocytes inseminated, mean fertilization rate was greater in the ICSI group when stratified by both age of the oocyte and sperm provider. The mean (SD) number of utilizable embryos per retrieval was similar between insemination techniques when stratified by both age of the oocyte and sperm provider (Table 3). The percentage of treatment cycles that reported no live birth was similar between insemination techniques (IVF: 72.25% vs. ICSI: 79.09%).

**Table 3:** Fertilization characteristics. January 1, 2013-December 31, 2022.

Fertilization and Embryology Outcomes		IVF		ICSI		*p*-Value
		Mean	SD	Mean	SD	
Oocytes inseminated/injected		10.67	7.49	8.40	6.16	<.0001
Normal Fertilization (2PN)	Oocyte Provider Age					
	0–34	7.61	5.77	7.85	5.76	0.0007
	35–37	6.38	5.19	6.33	4.97	0.5150
	38–40	5.43	4.61	5.36	4.44	0.2611
	41–42	4.60	3.93	4.45	3.84	0.0762
	43+	3.75	3.45	3.87	3.53	0.3325
	Sperm Provider Age					
	0–34	7.32	5.61	7.38	5.66	0.5100
	35–37	6.67	5.36	6.53	5.25	0.1342
	38–40	5.86	4.93	5.96	4.88	0.3183
	41–42	5.50	4.77	5.41	4.62	0.4394
	43+	5.07	4.54	4.93	4.34	0.1166
	Missing Data	6.20	5.26	6.21	5.03	0.9274
Fertilization rate (2PN/oocytes injected or inseminated)	Oocyte Provider Age					
	0–34	0.60	0.27	0.74	0.24	<.0001
	35–37	0.61	0.28	0.73	0.25	<.0001
	38–40	0.60	0.28	0.71	0.27	<.0001
	41–42	0.59	0.29	0.70	0.29	<.0001
	43+	0.59	0.31	0.69	0.30	<.0001
	Sperm Provider Age					
	0–34	0.60	0.27	0.73	0.24	<.0001
	35–37	0.61	0.27	0.73	0.26	<.0001
	38–40	0.60	0.28	0.72	0.26	<.0001
	41–42	0.58	0.29	0.70	0.28	<.0001
	43+	0.59	0.29	0.70	0.29	<.0001
	Missing Data	0.61	0.28	0.73	0.25	<.0001
	Embryo Recipient BMI					
	<18.5	0.67	0.22	0.75	0.21	<.0001
	18.5–25	0.64	0.23	0.75	0.22	<.0001
	25–30	0.63	0.23	0.76	0.22	<.0001
	30+	0.65	0.22	0.77	0.21	<.0001
	Missing Data	0.57	0.30	0.72	0.27	<.0001
Utilizable Embryo(s) per OPU	Oocyte Provider Age					
	0–34	4.99	4.66	4.90	4.43	0.1215
	35–37	4.14	3.99	4.01	3.82	0.0304
	38–40	3.40	3.29	3.33	3.23	0.1970
	41–42	2.87	2.62	2.66	2.67	0.0004
	43+	2.58	2.49	2.39	2.53	0.0241
	Sperm Provider Age					
	0–34	4.80	4.57	4.67	4.37	0.0505
	35–37	4.31	4.14	4.14	4.05	0.0195
	38–40	3.79	3.71	3.79	3.75	0.8968
	41–42	3.51	3.56	3.38	3.40	0.1664
	43+	3.25	3.27	3.07	3.16	0.0041
	Missing Data	3.95	3.88	3.74	3.58	0.0006
	Embryo Recipient BMI					
	<18.5	4.34	2.93	4.10	3.49	0.4021
	18.5–25	4.57	3.39	4.03	3.19	<.0001
	25–30	4.47	3.33	4.19	3.30	0.0027
	30+	4.45	3.28	4.13	3.22	0.0030
	Missing Data	3.85	4.36	3.82	3.90	0.4279

## Discussion

This large retrospective Canadian cohort study of patients undergoing IVF treatment for non-male factor infertility demonstrates a statistically significant benefit in cLBR when cIVF is compared to ICSI. Most published studies, to date, comparing ICSI to cIVF for non-male factor infertility have focused on fertilization rate and/or live birth rate as their primary outcome. Importantly, this is the largest, descriptive study with cLBR as the primary outcome and the first to report Canadian data across all ages in a non-male factor infertility population. The inclusion of cLBR as a primary outcome is unique as it incorporates fresh and frozen-thawed embryo transfers providing an indicator of success of an IVF cycle.

Our findings align with previous conclusions drawn from randomized trials (17, 18) and cohort studies from Latin America (9), Australia (10), China (11), and the USA (8) that failed to show improvements in cLBR when ICSI was used for a non-male factor infertility, despite being the most common method of insemination in Canada and worldwide (6, 19). In their retrospective analysis of 49,813 cycles, Schwarze et al. (9) showed that live birth rate per cycle was lower in the ICSI group (22.99% vs. 28.76%). In 2018, Li (10) demonstrated similar cLBR for IVF (37%) and ICSI (36%) in 21,072 embryo transfer cycles. In a sub-analysis of a single center retrospective cohort, Liu et al. (11) reported a similar cLBR to ours of 41%, defined as a live delivery at ≥28 weeks, for IVF and ICSI cycles with non-male factor infertility who underwent their first fresh cycle embryo transfer. Recently, Iwamoto et al. (8) reported on 46,967 first autologous IVF cycles with non-male factor infertility and showed no difference in cLBR between IVF (64.3%) and ICSI (60.9%) in cycles without PGT-A. They defined cLBR as all associated fresh and linked frozen transfer cycles from a single retrieval cycle up to the first live birth and calculated cLBR on a per patient basis.

Our results align with previous reports (8–11) demonstrating that ICSI is the most prevalent mode of insemination. In our study, ∼75% of cycles used ICSI. The differences in cLBR observed between ours and other studies may be due to regional practices differences, variation in laboratory techniques, differences in the inclusion/exclusion criteria, the inclusion of fresh and frozen transfers, and how cLBR is defined and calculated. While a standardized definition is encouraged (16) nuance in the calculation can effect results. For instance, including more than one IVF cycle from the same couple, extending the time interval to achieve pregnancy by including all subsequent frozen transfers until a live birth is achieved, calculating cLBR on a *per patient* rather than a *per cycle* basis, and including a wider range of dates in study parameters, as we did in the current study, may have resulted in the observed differences in cLBR.

It is well established that improvements in technology, laboratory practice and technique have changed over time resulting in better patient outcomes and this may have resulted in temporal differences. In addition, including cycles from as early as 2013 may have resulted a ‘drag effect’ pulling the average cLBR down. Finally, calculating cLBR on a per patient basis rather than a per cycle basis can inflate the cLBR observed. For example, in the CARTR annual data audit report (14) when cLBR is calculated for all *cycles* the overall mean cLBR is 39% and when calculated on a per *patient* basis the cLBR is 48%; a difference of ∼10% and more inline with recent data published by the Society for Assisted Reproductive Technology (SART) clinics (8) Nonetheless, our reported cLBRs align with previously reported data from CARTR (14).

In patients with non-male factor infertility, it has previously been hypothesized (8) that the lack of benefit of ICSI over cIVF may be due to the necessity of injecting only mature oocytes with ICSI thereby limiting the pool of available oocytes for insemination. Concerns around the ICSI technique itself may partly account for differences between the two insemination methods. For instance, the invasive nature of ICSI circumvents natural selection mechanisms allowing the most suitable sperm to fertilize an ovum bypassing several crucial steps in the fertilization process (20). Additional concerns of plausible risks associated with ICSI have been previously raised including chromosomal, congenital and anatomic anomalies. ICSI has been shown to compromise nuclear decondensation of spermatozoa leading to possible embryo aneuploidy (21), disrupt the egg meiotic spindle leading the chromosomal segregation abnormalities (22), and the increased handling of oocytes outside the incubator may alter the temperature and pH which increases the rates of stress-induced pathology (23). Although most of these findings are reported in studies with abnormal semen parameters and thus may be related to the underlying male factor, the biological plausibility should not be overlooked. Nonetheless, our study reports similar cLBR between cIVF and ICSI in a non-male factor population that aligns with others reported in the literature (10, 11) and the most recent annual summary of all CARTR data (19).

When looking at fertilization rate, defined as the number of 2PN divided by the total oocytes inseminated, there were no clinically relevant differences by age of egg or sperm provider when looking at the data within insemination technique. This finding is supported by previous reports demonstrating that rates of normal fertilization were unaffected by the increasing age of either partner (24). However, when comparing the fertilization rate between insemination technique fertilization rate was higher for ICSI when compared to cIVF (70% vs. 60%), a finding that aligns with previous studies (10) and guidelines (2) and may be attributable to the fact that more immature oocytes are inseminated with IVF.

A unique aspect of our dataset is the inclusion of a variable highlighting the number of utilizable embryos per retrieval. This was defined as the number of embryos utilizable by the end of the culture period, regardless if surplus embryos were frozen. Overall, we demonstrated a decrease by age of oocyte provider (4.9 to 2.4) a finding likely attributable to the natural decline in fecundity with advanced reproductive age. We also demonstrated similar rates of utilizable embryos between IVF and ICSI which aligns with previous reports (8).

A limitation of this study is the retrospective and descriptive nature of the analysis rendering us unable to make causal inferences and account for potential confounding due to baseline variable differences. However, many of these statistical differences (e.g., baseline age) were clinically similar and unlikely to influence the results. As with any large dataset inherent issues with variable availability, data input and acquisition are common. Like our American colleagues using SART data, there is no set definition of male factor infertility in the CARTR database. It is the responsibility of individual clinics to correctly diagnose this subcategory of infertility, which is commonly done using the WHO criteria (15).

Furthermore, we are unable to provide outcome data on the cohort excluded from the analysis (Figure 1) and this may limit generalizability of the findings. We are also unable to establish a clear link between birth outcomes based on whether PGT-A testing was performed as this variable was not available in the dataset. Furthermore, we acknowledge that the difference in the ‘intent to perform PGTA’ variable in the ICSI group may contribute to selection bias and is a notable weakness. Nonetheless, this finding likely reflects earlier recommendations that required ICSI with PGT-A, particularly when older analytical platforms were employed. However, given that our results align so closely with recent publications, including that from the USA (8) where cLBR did not differ between groups whether PGT-A was used or not, adds strength to our paper. Similarly, there is no demographic data collected on several important variables including ethnicity, gender identity and/or household income. The later limits the generalizability of our findings to other regions outside of Canada where the population may differ. Given that we were provided with aggregate data, we were unable to calculate total fertilization failure on an individual level. Despite this, total fertilization failure is uncommon in non-male factor infertility and while the routine use of ICSI may decrease the incidence of unexpected total failed fertilization, current guidelines suggest more than 30 couples would have to undergo ICSI unnecessarily to prevent one case of total failed fertilization (2). However, we do present fertilization rates, which we showed to be higher with ICSI, and in line with existing data given the fewer number of oocytes injected.

Limitations of the CARTR-BORN data reporting system preclude knowing how many embryos were transferred (i.e., 1 vs. 2) in our cohort which may be partially responsible for differences in cLBR between studies. Finally, we received data in aggregate and not on the individual patient level, thus regression models were not performed. This limits our ability to adjust for potential confounding or selection bias and may contribute to the observed results between cIVF and ICSI in this population. However, our large sample size should mitigate this to a degree.

Despite these shortcomings, it is important to recognize that the CARTR Plus database has been previously tested and validated (13). And more importantly, our results align with previous randomized controlled trials (17, 18) and retrospective cohort studies demonstrating no benefit of ICSI in a non-male factor infertility population (8–11). Collectively, despite these shortcomings our large, first of its kind study utilizing validated national registry data mirrors previous reports and complements existing studies providing a valuable contribution to the literature.

Future work in this area should be prospective in nature, employ a design that utilizes sibling oocytes inseminated by both techniques, be able to report on whether PGT testing was used, and calculate fertilization rate by number of oocytes retrieved as this would provide a more accurate estimate based on insemination technique. Furthermore, acquiring individual patient level data and completing a regression analysis to address potential confounding would be valuable. From a clinical perspective, qualitative studies are needed to evaluate reproductive medicine specialist rationale for choosing ICSI in the absence of male factor and understand why they continue to choose this insemination method when no long-term benefits have been demonstrated in cLBR or live birth rate, in cohort and randomized trials, respectively. Incorporating sociodemographic variables including race, ethnicity, and socio-economic status may further shed light on individuals seeking infertility treatments and make results generalizable to diverse populations. Furthermore, research looking at the impact of government funded IVF may also provide insight into clinical decision-making and patient counselling.

## Conclusion

The use of ICSI in non-male factor infertility does not improve cLBR when compared to cIVF in this large Canadian retrospective cohort. Here we present the largest number of IVF cycles in a non-male factor infertility population and are the first Canadian study on this topic. Our ability to calculate cLBR provides a significant addition to the growing body of evidence suggesting the lack of benefit in using ICSI in this population. In our analysis, we were able to include cycle details, including markers of ovarian reserve, medication information, and follicle details from cycle monitoring, that are not previously reported in other retrospective cohorts. Although markers of ovarian reserve do not predict live birth (25), it was reassuring to note that AMH, total FSH dose, estradiol and progesterone levels on trigger day, and the number of small <15 mm and large >15 mm follicles at trigger day were similar between groups and is an important addition to the literature.

Although the use of ICSI far exceeds the number of patient(s) presenting with abnormal semen parameters, the choice to use ICSI as an insemination technique ultimately comes down to a decision that is made between the patient and their provider. Having difficult conversations around poor or total fertilization failure following cIVF, despite normal semen parameters may encourage some patients and/or providers to favor the routine use of ICSI. Nonetheless, the patient's reproductive autonomy should be at the forefront to ensure that they feel supported and informed of the risks, benefits, and alternatives to employing ICSI in cases of non-male factor infertility.

## Data Availability

Requests to access these datasets should be directed to the corresponding author as this data is not available to the public.
